# Physical interpretation of nonlocal quantum correlation through local description of subsystems

**DOI:** 10.1038/s41598-022-17540-1

**Published:** 2022-09-30

**Authors:** Tanumoy Pramanik, Xiaojiong Chen, Yu Xiang, Xudong Li, Jun Mao, Jueming Bao, Yaohao Deng, Tianxiang Dai, Bo Tang, Yan Yang, Zhihua Li, Qihuang Gong, Qiongyi He, Jianwei Wang

**Affiliations:** 1 State Key Laboratory for Mesoscopic Physics, School of Physics, Peking University, Beijing, 100871 China; 2 Beijing Academy of Quantum Information Sciences, Beijing, 100193 China; 3 Institute of Microelectronics, Chinese Academy of Sciences, Beijing, 100029 China; 4 Frontiers Science Center for Nano-optoelectronics, Collaborative Innovation Center of Quantum Matter, Peking University, Beijing, 100871 China; 5 Collaborative Innovation Center of Extreme Optics, Shanxi University, Taiyuan, 030006 Shanxi China; 6 Peking University Yangtze Delta Institute of Optoelectronics, Nantong, 226010 Jiangsu China

**Keywords:** Quantum information, Qubits

## Abstract

Characterization and categorization of quantum correlations are both fundamentally and practically important in quantum information science. Although quantum correlations such as non-separability, steerability, and non-locality can be characterized by different theoretical models in different scenarios with either known (trusted) or unknown (untrusted) knowledge of the associated systems, such characterization sometimes lacks unambiguous to experimentalist. In this work, we propose the physical interpretation of nonlocal quantum correlation between two systems. In the absence of *complete local description* of one of the subsystems quantified by the *local uncertainty relation*, the correlation between subsystems becomes nonlocal. Remarkably, different nonlocal quantum correlations can be discriminated from a single uncertainty relation derived under local hidden state (LHS)–LHS model only. We experimentally characterize the two-qubit Werner state in different scenarios.

## Introduction

**Figure 1:**
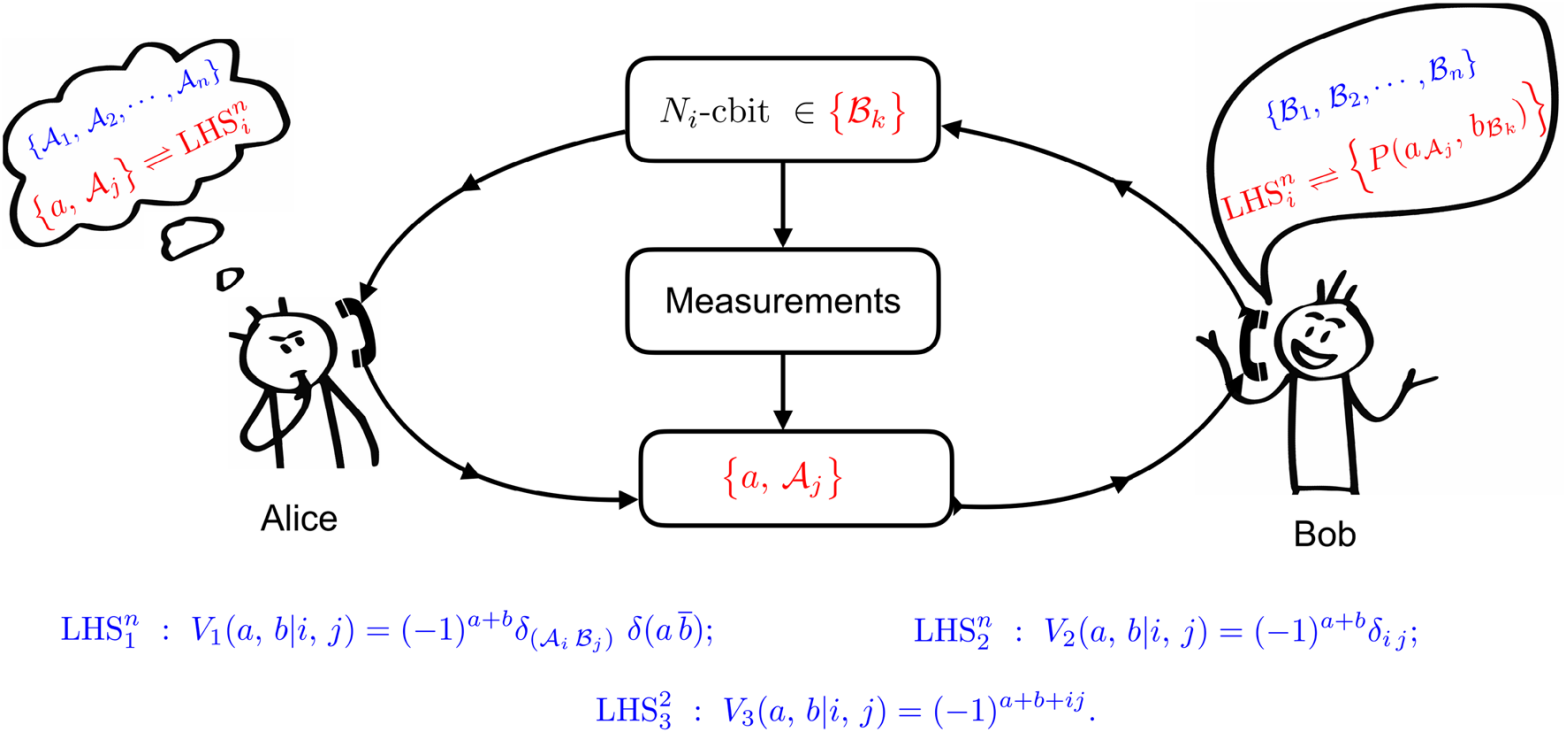
Characterization of different nonlocal quantum correlations of the shared state $\rho _{AB}$ in a single local-description model. Bob’s task is to characterize quantum correlations by the violations of the local-uncertainty relations. $\text {LHS}_i^n$, $i=1,2,3$, refers to different Bob’s strategies in different scenarios to verify nonlocal correlations including entanglement, steerability and Bell nonlocality. *n* is the number of measurement. Bob first asks Alice to minimize his uncertainty about the state of the system *B* by communicating $N_i$-cbits (classical bits) to Alice. Alice then measures the appropriate observable ${\mathcal {A}}_j$ on the system *A* and communicates the $\{a,\,{\mathcal {A}}_j\}$ information back to Bob. Given the $\{a,\,{\mathcal {A}}_j\}$ information, Bob checks the uncertainty of the state of his system *B*. If the certain local-uncertainty relation is violated as Eq.(2) shown, Bob confirms that the shared state $\rho _{AB}$ is either entangled, steerable, or Bell nonlocal. The figure is taken form the source https://www.dreamstime.com/ and then it has been modified for the present scenarios.

Quantum correlation between two or more subsystems that cannot be described by local-causal theories is a key resource in quantum information science^1–20^. A crucial task is to characterize, categorize and certificate different quantum correlations. In general, quantum correlations can be described by the joint probability distribution of the events measured in the subsystems. For the bipartite quantum systems, the correlation is defined by

1 $$\begin{aligned} {\mathcal {P}}=\left\{ P(a_{{\mathcal {A}}_i}, b_{{\mathcal {B}}_j}| \rho _{AB}) = \text {Tr}\left[ \left( \Pi ^{{\mathcal {A}}_i}_a\otimes \Pi ^{{\mathcal {B}}_j}_b\right) \rho _{AB}\right] \right\} \,\,\, \end{aligned}$$

where $\rho _{AB}$ is the unknown state composed by Alice's and Bob's systems, and $\Pi ^{{\mathcal {A}}_i}_a$ ($\Pi ^{{\mathcal {B}}_j}_b$) is the projective measurement having outcomes of *a* (*b*) for the ${\mathcal {A}}_i$ (${\mathcal {B}}_j$) observable. The characterization of correlation of the state $\rho _{AB}$ implies the measurement of the probability distribution ${\mathcal {P}}$. For example, to certify the Bell nonlocality, the distribution ${\mathcal {P}}$ has to violate Bell inequalities^2,5,21^. Quantum correlations are further categorized by entanglement^6^ and quantum steering^3,7,22^. Wiseman et al. proposed a framework to describe all the three quantum correlations for the bipartite system by considering three different scenarios having either known (trusted) or unknown (untrusted) knowledge of the system^7,8,23^: (1) $\rho _{AB}$ is entangled if ${\mathcal {P}}$ can not be generated by a separable state having trusted measurement devices in both subsystems. (2) $\rho _{AB}$ is steerable if ${\mathcal {P}}$ can not be produced by a local hidden state (LHS) model, in the case that one subsystem owns trusted measurement device while the other remains untrusted. (3) $\rho _{AB}$ is Bell nonlocal if ${\mathcal {P}}$ is incompatible with the local hidden variable (LHV) interpretation and both measurement devices are untrusted. Categorizing quantum correlations regarding their capability of controlling measurement apparatuses have enabled important applications in quantum information, e.g., device independent (DI) or one-side DI quantum key distribution^24–26^ and randomness generation^27^. We however note that non-separability, steerability, and Bell nonlocality can only be verified by the violations of their own inequalities, asking for a general framework of characterizing quantum correlations. The conceptual definition of known or unknown systems may also lead to confusion and ambiguity to experimentalist who usually can well control the system and measurement apparatuses (Fig. 1).

**Figure 2:**
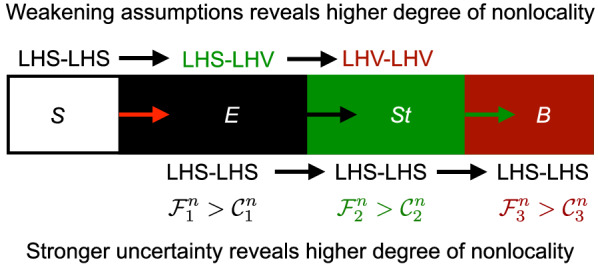
*S*, *E*, *St*, and *B* correspond to separable, entangled, steerable, and Bell nonlocal correlation, respectively. Entanglement, steerability, and Bell nonlocal correlation are confirmed if the observed correlation ${\mathcal {P}}$ of Eq. (1) can not be explained by the theoretical models LHS–LHS, LHS–LHV, and LHV–LHV, respectively. Less assumption about the associated systems makes the correlation more nonlocal. In this work, we discriminate the degree of nonlocality under a single theoretical model, LHS–LHS with the help of proposed uncertainty relation of inequality (2). Here, the violation of the inequalities ${\mathcal {F}}_1^n\le {\mathcal {C}}_1^n$ (5), ${\mathcal {F}}_2^n\le {\mathcal {C}}_2^n$ (3) and ${\mathcal {F}}_3^n\le {\mathcal {C}}_3^n$ (4) validates entanglement, steerability and Bell nonlocal correlation, respectively.

In this work, we propose a more physical interpretation of different nonlocal quantum correlations from *complete local description* of the subsystems that can be quantified by the *local uncertainty relation* of the subsystems. Our idea is inspired by the Einstein’s comment^1^ and Bell’s seminal work^2^ on incompleteness of quantum theory supplemented by LHV. We here ask a similar question: *when two systems A and B are quantumly correlated, is there any complete local description of one of the subsystems, say, B has nothing to do with A, or vice versa*? We will show how the local uncertainty relation derived using the complete local description of subsystems can help in discriminating different nonlocal quantum correlations. We remark that our way of characterizing quantum correlations represents the fundamental connection of quantum nonlocality and uncertainty relation. Note that, in the previous works^2,5,7,8,21,23,28,29^, the criteria of on discrimination of different non-local quantum correlations are based on different forms of uncertainty relation formulated under LHS-LHS, LHS-LHV, LHV-LHV model. Here, we introduce single uncertainty relation (inequality (2)) formulated under LHS-LHS model, and this uncertainty relation can discriminate three different kinds of nonlocal correlations, e.g., entanglement, steering, Bell nonlocal correlation. See the Fig. (2) for more clear picture.

## Verification of different nonlocal correlations through complete description of subsystems

Let us first consider the following game. Alice prepares a joint system of *A* and *B* in an unknown state $\rho _{AB}$, and sends the subsystem *B* to Bob. While Bob may think that Alice can cheat him by preparing the state according to the LHS model, $\rho _{AB}^{\text {LHS}} = \sum _{i} p_i \rho ^A_i\otimes \rho ^B_i$, where $\rho ^A_i$ ($\rho ^B_i$) is Alice’s (Bob’s) local state, $p_i\ge 0$ and $\sum _{i} p_i=1$. For $\rho _{AB}^{\text {LHS}}$, the system *B* has complete local description, $\{p_i,\,\rho ^B_i\}$. Here Bob tends to characterize the nonlocal quantum correlations of the state $\rho _{AB}$ with the help of the local uncertainty relation. Bob asks Alice to minimize the uncertainty of the state of system *B* by communicating *k*-cbit (classical bit) information. Given the *k*-cbit, Alice measures an observable of ${\mathcal {A}}_i$, and sends back the measurement outcome *a* together with ${\mathcal {A}}_i$ to Bob. Finally, Bob checks whether the joint probability distribution ${\mathcal {P}}$ can be describe by the complete local description of $\rho _{AB}^{\text {LHS}}$. This description is certified by the uncertainty of their outcomes characterized by the condition *V*(*a*, *b*|*i*, *j*) (where *i*, *j* represent Alice’s and Bob’s choice of observables $\{{\mathcal {A}}_i\}$ and $\{{\mathcal {B}}_j\}$, respectively). Bob can confirm that the state $\rho _{AB}$ is entangled if the following local uncertainty relation is violated

2 $$\begin{aligned} {\mathcal {F}}^n_k = \bigg \{\sum _{i,j=0}^{n-1} \sum _{a,b=0}^{1} V_k(a,b|i,j) P(a_{{\mathcal {A}}_i},\,b_{{\mathcal {B}}_j}| \rho _{AB}) \bigg \} \le {\mathcal {C}}^n_k, \,\,\,\,\, \end{aligned}$$

where $V_k(a,b|i,j)$, $k\in \{1,2,3\}$, represents three different conditions of quantum correlations ($V_1$ for entanglement, $V_2$ for steering, and $V_3$ for Bell nonlocality); where *n* is the number of measurement performed on *A* and *B* and chosen as $n=2,3$ in our work (also in the experiment), which however can be chosen to an arbitrary number (see the ﻿Supplementary Materials). The upper bound ${\mathcal {C}}^n_k$ is obtained by maximizing ${\mathcal {F}}^n_k$ over the state of $\rho _{AB}^{\text {LHS}}$ and Alice’s all possible strategies. The violation of inequality (2) implies that the shared state $\rho _{AB}$ cannot be written in the form of $\rho _{AB}^{\text {LHS}}$.

Figure 1 sketches three different scenarios for the characterization and certification of quantum correlations in a single local-description model. For simplicity, we start with the $LHS_2^n$ one, that is the LHS model description for quantum steering^4,7,8,30^).

### Verification of steerability

For the verification of steerability of the shared state $\rho _{AB}$, Bob asks to minimize his uncertainty of observables ${\mathcal {B}}_i$. He checks the uncertainty of their outcomes constrained by the condition of $V_2(a,b|i,j)=(-1)^{a+b} \delta _{ij}$, and the local uncertainty relation thus turns into^31,32^

3 $$\begin{aligned} \bigg \{{\mathcal {F}}_2^n=\sum _{i=0}^{n-1}|\langle {\mathcal {A}}_i\,{\mathcal {B}}_i\rangle |\bigg \} \le \bigg \{C_2^n=\max _{\{{\mathcal {A}}_i\},\rho _{AB}^{\text {LHS}}}\left[ {\mathcal {F}}_2^n\right] \bigg \}, \end{aligned}$$

where the upper bound, $C_2^3 = \sqrt{3}$ ($C_2^2=\sqrt{2}$) for $n=3$ ($n=2$) measurement setting corresponds to the local description of Bob’s system by the eigenstates of the observables $(\sigma _x\pm \sigma _y\pm \sigma _z)/\sqrt{3}$ ($(\sigma _x\pm \sigma _z)/\sqrt{2}$) (see Supplementary Materials for details). The $V_2$ shown as Eq. (3) represents Bob’s residual uncertainty of the observable ${\mathcal {B}}_i$ (randomly chosen from a set of non-commuting observables^33–35^), given the $\{a,\,{\mathcal {A}}_i\}$ information from Alice. The classical communication of 1-cbit ($\log _2^{n=2}$) or 1.58-cbit ($\log _2^{n=3}$) is required from Bob to Alice when Bob randomly chooses ${\mathcal {B}}_i$ from a set of $n=2$ or 3 observables, say, $\{\sigma _z,\,\sigma _x\}$ or $\{\sigma _z,\,\sigma _x,\,\sigma _y\}$, respectively. The violation of inequality (3) indicates that the system *B* does not have complete local description independent of the system *A*, and the correlation is known as quantum steering^31,32,36^.

### Verification of Bell nonlocal correlation

For the verification of Bell nonlocal, Bob does not reveal the choice of observables and there is no communication from Bob to Alice. Given information from Alice, Bob estimates the uncertainty from the measured probability distribution ${\mathcal {P}}$. In the case of $n=2$ measurement, the uncertainty of input $\{i,j\}$ and output $\{a,b\}$ correlation is determined by the CHSH game($V_3(a,b|i,j)=(-1)^{(a+b+ij)}$ which corresponds to the winning condition of the Clauser-Horne-Shimony-Holt game)^2,21,37^. Thus, the local-uncertainty Eq. (2) can be rewritten as

4 $$\begin{aligned} {\mathcal {F}}_3^2 \le \bigg \{\max _{\{{\mathcal {A}}_i\},\,\rho _{AB}^{\text {LHS}}}\left[ {\mathcal {F}}_3^2\right] = 2\bigg \}, \end{aligned}$$

where ${\mathcal {F}}_3^2=|\langle {\mathcal {A}}_0\left( {\mathcal {B}}_0+{\mathcal {B}}_1\right) \rangle + \langle {\mathcal {A}}_1\left( {\mathcal {B}}_0-{\mathcal {B}}_1\right) \rangle |$ and the upper bound corresponds to local description of Bob’s system by the state, e.g., $|0\rangle$. The $V_3$ corresponds to Bob’s residual uncertainty of the randomly chosen observables of $\{{\mathcal {B}}_0=\sigma _x,\,{\mathcal {B}}_1=\sigma _z\}$^33,38^, with respect to Alice’s individual measurement from $\{{\mathcal {A}}_0, {\mathcal {A}}_1\}$. When the local uncertainty relation (4) is violated, Bob validates the Bell nonlocal correlation^2,21,37^. The inequality (4) becomes a necessary and sufficient condition for Bell nonlocality for the 2-measurement settings and binary outcomes^39^. Note that the $\text {LHS}_3^2$ model is a stricter version of $\text {LHS}_2^n$ model, as the former represents a simultaneous steerability (uncertainty) of $\{{\mathcal {B}}_0,\,{\mathcal {B}}_1\}$^37^ while the later represents an individual steerability (uncertainty) of ${\mathcal {B}}_i$ with respect to Alice’s observable ${\mathcal {A}}_i$. Therefore, the Bell nonlocal correlation becomes the strongest form of nonlocal correlations – the violation of inequality (4) indicates the violation of inequality (3).

### Verification of entanglement

To certify entanglement, Bob asks to minimize the value of *b* for the ${\mathcal {B}}_j$ measurement, randomly chosen from the set of non-commuting observables. The classical communication of 2-cbit (four possible combinations of $\{a,{\mathcal {B}}_j\}$) or 2.58-cbit (six possible combinations) is required from Bob to Alice, when $n=2$ or 3-measurement is chosen, respectively. Bob evaluates the uncertainty of $V_1(a,b|i,j)=(-1)^{a+b} \delta _{({\mathcal {A}}_i{\mathcal {B}}_j)} \delta _{(a{\overline{b}})}$, where ${\overline{b}}=b\oplus 1$. Applying the condition of $V_1$ in the inequality (2), the local uncertainty relation turns into

5 $$\begin{aligned} \bigg \{{\mathcal {F}}_1^n= & {} \sum _{i=0}^{n-1} P({\mathcal {A}}_i,\,{\mathcal {B}}_i)\bigg \} \le \bigg \{\max _{\rho _{AB}^{\text {LHS}}}\left[ {\mathcal {F}}_1^n\right] = {\mathcal {C}}_1^n\bigg \} \end{aligned}$$

where $P({\mathcal {A}}_i,\,{\mathcal {B}}_i)=P(0_{{\mathcal {A}}_i},\,1_{{\mathcal {B}}_i}) + P(1_{{\mathcal {A}}_i},\,0_{{\mathcal {B}}_i})$, 0 and 1 refer to measurement outcomes; where ${\mathcal {C}}_1^{n=3}=2$, ${\mathcal {C}}_1^{n=2}=1$ corresponds to Bob’s local state, e.g., $|0\rangle$ (see details in Supplementary Materials). The $V_1$ leads to the uncertainty of anti-correlated outcomes $a\oplus b=1$ when Alice and Bob both performs measurement of the same observable, i.e, ${\mathcal {A}}_i={\mathcal {B}}_j$ on their respective subsystems. The violation of the local uncertainty relation in Eq.(5) can confirm the presence of entanglement^28,40–42^. The uncertainty relation of Eq. (5) is the weaker form of Eq. (3), as quantum steering considers uncertainty of all possible combinations of $\{a, b\}$, while entanglement only takes uncertainty of anti-correlated outcomes.

**Figure 3:**
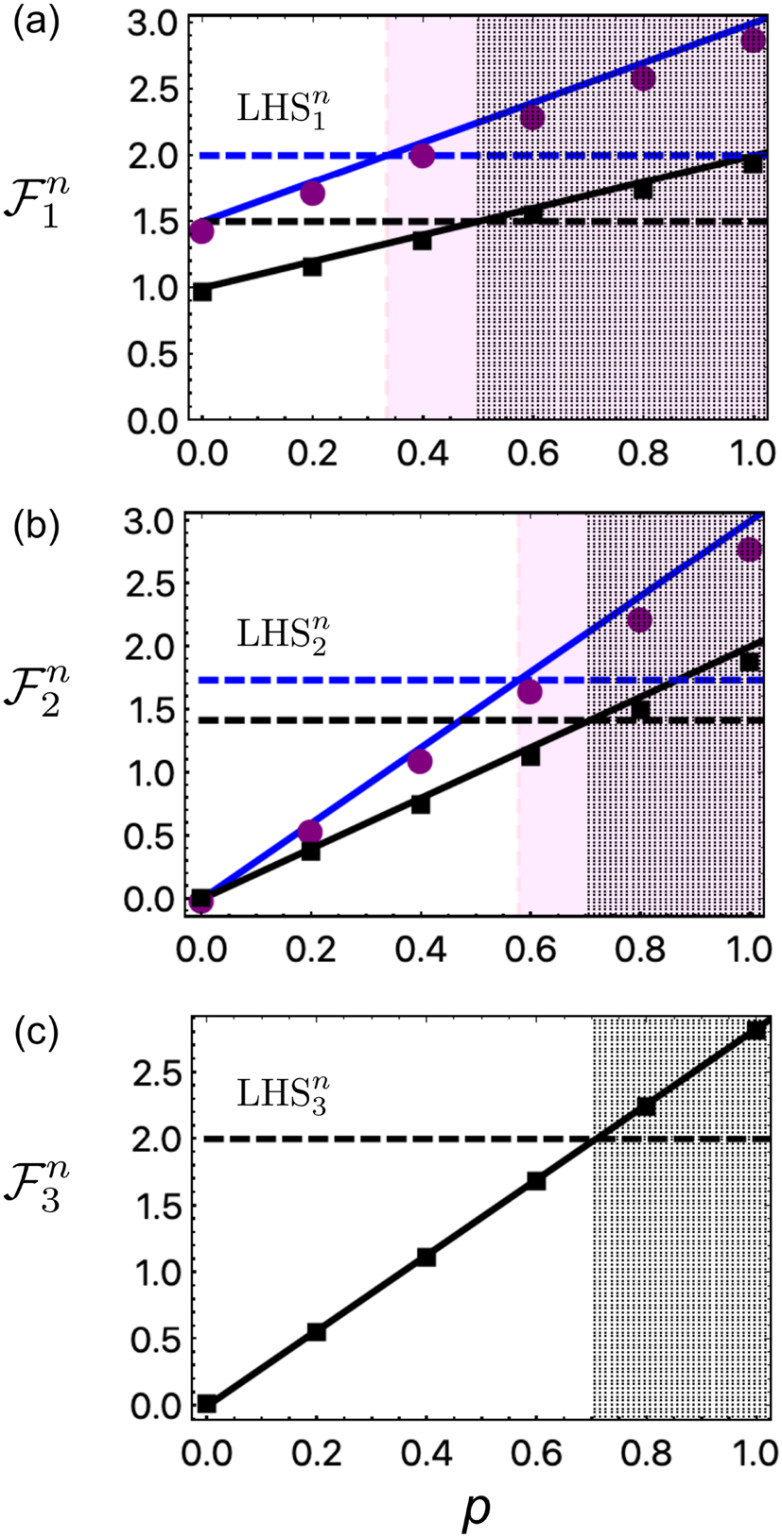
Theoretical and experimental characterizations of (**a**) entanglement, (**b**) steerability, and (**c**) Bell nonlocality for the bipartite Werner state. The $\text {LHS}_i^n$, $i=1,2,3$ and $n=2,3$, refers to different LHS models in the three scenarios, see the derived local uncertainty relations of (3–5). All experiments were implemented on an integrated silicon-photonics quantum device. Points denote experimental data and lines denote theoretical prediction: circular and square points are for $n=3$ and $n=2$ measurement settings; blue and black lines are for $n=3$ and $n=2$ measurement, respectively. Red shaded (black dotted) regime in (**a**–**c**) identifies the *p* mixing parameter of the Werner state $\rho _W$, above which the state is certified as entanglement, steerable, and Bell nonlocal, for $n=3$ ($n=2$) measurement settings, respectively. Horizontal dashed lines are plotted for the guidance the achievable upper bound of the inequality value, ${\mathcal {F}}^n_k$. Note error bars ($\pm \sigma$) estimated from 20 sets of data are too small to be invisible in the plot.

**Figure 4:**
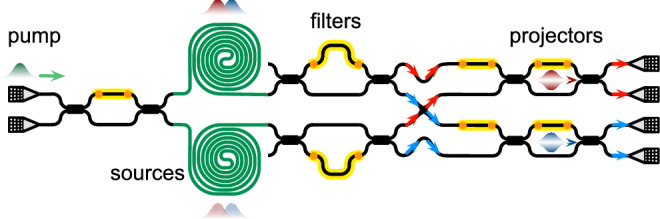
Schematic of an integrated silicon-photonics quantum device. The quantum device is capable of generating, manipulating and analyzing maximally path-entangled states. The device is fabricated on the silicon-on-insulator platform. Lines are silicon nanophotonic waveguides with the size of 450 nm $\times$ 220 nm, and yellow parts are thermo-optic phase shifters that can be precisely controlled in experiment. A continuous wave laser light (at the wavelength of 1550.12 nm) was used to pump two photon-pair sources, producing a pair of path-entangled photons via the spontaneous four-wave mixing (sFWM) process. The entangled photons were locally manipulated and analyzed by Alice (signal photon at 1545.31 nm) and Bob (idler photon at 1554.91 nm), respectively, which were implemented by the terminate Mach-Zehnder interferometers (MZIs). The two photons are measured by two superconducting nanowire single-photon detectors (SNSPDs), and their coincidence were recorded by a time tagger.

###  Higher degree of nonlocality from more uncertainty of the condition $V_k(a,\,b|i,\,j)$

For the purpose of connecting uncertainty relation (2) with the degrees of nonlocality, we define normalized probability by ${\mathcal {P}}_k^n={\mathcal {F}}_k^n/\max \left[ {\mathcal {F}}_k^n\right]$, where $\max \left[ {\mathcal {F}}_k^n\right]$ corresponds to algebraic maximum of ${\mathcal {F}}_k^n$. The corresponding uncertainty is measured by the Shannon entropy, ${\mathcal {H}}_k^n= - {\mathcal {P}}_k^n \log 2\left[ {\mathcal {P}}_k^n\right] - (1-{\mathcal {P}}_k^n) \log 2\left[ 1-{\mathcal {P}}_k^n\right]$. Therefore, ${\mathcal {H}}_k^n$ determines the degree of uncertainty of the event $V_k(a,\,b|i,\,j)$, which corresponds to the correlation between Alice’s and Bob’s outcomes *a* and *b*. For entanglement, steerability and Bell nonlocal correlation ${\mathcal {H}}_1^3 > 0.92$ (corresponds to the inequality (5), ${\mathcal {F}}_1^3 > 2$ and $\max \left[ {\mathcal {F}}_1^3\right] =3$), ${\mathcal {H}}_2^3 > 0.98$ (corresponds to the inequality (3), ${\mathcal {F}}_1^3 > \sqrt{3}$ and $\max \left[ {\mathcal {F}}_2^3\right] =3$), ${\mathcal {H}}_3^2 > 1$ (corresponds to the inequality (4), ${\mathcal {F}}_1^3 > 2$ and $\max \left[ {\mathcal {F}}_3^2\right] =4$), receptively. As a result, higher degree of nonlocality implies larger threshold value of uncertainty of the condition $V_k(a,\,b|i,\,j)$.

Our local uncertainty relations as shown by inequalities of (3)–(5), which are all derived from a single inequality of (2) under different conditions, represent the more physical interpretation of different quantum correlations including quantum entanglement, steering and Bell nonlocal correlation. We now take the Werner state of $\rho _W= p\,\rho _{|\phi ^-\rangle } + (1-p)\,\frac{{\mathrm{I}}\otimes {{\mathrm{I}}}}{4}$ as an example to test our local-description model in theory and experiment. $\rho _{|\phi ^-\rangle }$ is the density matrix of the singlet state of $|\phi ^-\rangle =(|01\rangle - |10\rangle )/\sqrt{2}$, *I* is the identity matrix, and *p* ($0\le p\le 1$) denotes the mixing parameter. The task now is to determine both in theory and experiment the bound of the *p* parameter, above which the inequalities (3)–(5) can be violated and thus the state $\rho _W$ can be certified to be entangled, steerable, or Bell nonlocal. The results are shown in Fig. 3.

## Experimental demonstration of different nonolocal correlations

We experimentally verified the three quantum correlations for the Werner state. Figure 4 shows the diagram of an integrated silicon-photonic quantum device that can generate, manipulate and analyze all four Bell states^43,44^. The integrated quantum device offers high levels of controllability and stabilities of operating quantum states of light^45,46^. The maximally entangled state has been created with a high fidelity of $0.951\pm 0.096$ by performing quantum state tomography (QST). The experimental realization of the $\rho _W$ state with a fully controllable mixture parameter *p* is enabled by the classical mixture of quantum states (see experimental details in Supplementary Materials).

**Figure 5:**
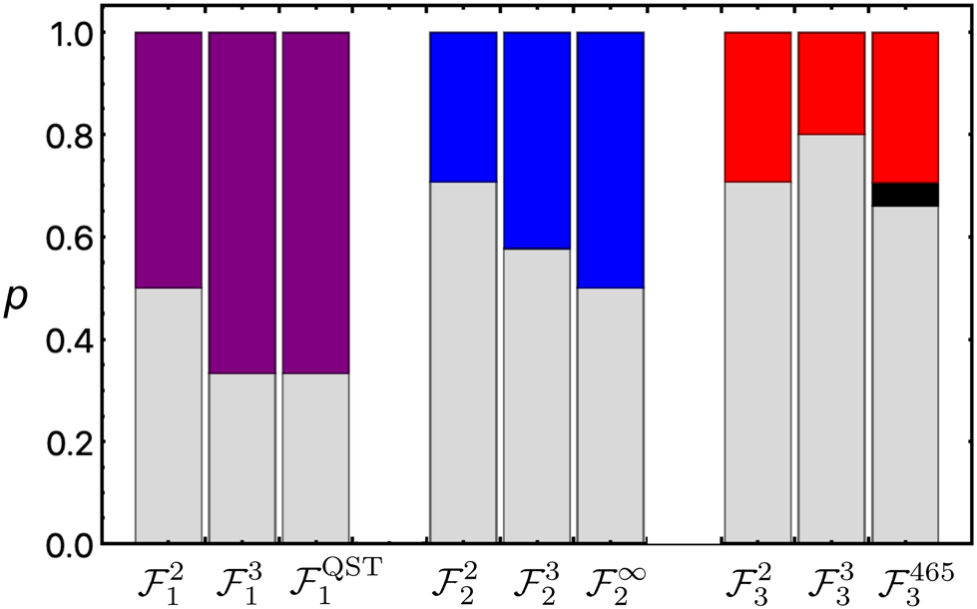
Bound of inequality violation for the quantum correlations of entanglement (${\mathcal {F}}^n_1$), steerability (${\mathcal {F}}^n_2$) and Bell nonlocality (${\mathcal {F}}^n_3$). The number of measurement settings $n=2,3$ are considered, while the ${\mathcal {F}}^{\infty }_k$ value is estimated from infinite measurement settings, and ${\mathcal {F}}^{\text {QST}}_k$ is quantified by QST. Purple, blue and red colored regime represents the bound of the *p* mixture parameter, above which the state is certified as entanglement, steerable, and Bell nonlocal, respective. Grayed regimes denote the presence of LHS model. Note the blacked regime refers to the inconclusive regime for Bell nonlocality.

Figure 3 shows the characterizations of entanglement, steering and Bell nonlocal, experimentally demonstrating the violations of their inequalities of (5), (3) and (4), respectively. In Fig. 3a, for $n=2$ and 3-measurement settings, entanglement is confirmed for $1/2<p\le 1$ (black dotted) and $1/3<p\le 1$ (red shaded), respectively^28^. Note that 3-measurement is sufficient to fully reveal entanglement of the $\rho _W$ state up to the value obtained by QST (see Supplementary Materials and Fig. 5). In Fig. 3b, quantum steerability is certified when $1/\sqrt{3}<p\le 1$ (red shaded) for the 3-measurement setting, larger than that for the 2-measurement setting having $1/\sqrt{2}<p\le 1$ (black dotted). Increasing the number of measurement of *n* can relax the *p* value of demonstrating steering^31,32^. For example, when implementing infinite measurement settings, the steerability inequality can be violated for $1/2<p\le 1$^8^. In Fig. 3c, it shows that the state is demonstrated to be Bell nonlocal for $0.7071<p\le 1$. Unlike the steering and entanglement scenarios, increasing the number of measurement to three however does not relax the choice of *p* parameter. Bell nonlocality can be verified for $4/5<p\le 1$ using the $I_{3322}$ inequality, as reported in Ref.^47^.

Figure 5 summarizes the bound of violating the LHS inequalities for entanglement, steering and Bell nonlocal. We here consider the ${\mathcal {F}}^n_k$ for the $n=2,3$ measurement settings, and for infinite measurements and for QST measurement. The regimes of *p* parameter obeying the LHS models are grayed, while the regimes for certificated entanglement, steerability, and Bell nonlocality are colored. In the ${\mathcal {F}}^{465}_3$ bar, the red regime was estimated with 465 measurement settings^48^, and the black one refers to an unknown regime^49^.

## Conclusions

In sum, we formulate single uncertainty relation under LHS-LHS model and different kinds of nonlocal correlations can be discriminated through it. This is major improvement over previously used different uncertainty relation based on different theortical models, e.g., LHS-LHS for entanglement, LHS-LHV for steering and LHV-LHV for Bell nonlocal correlation. We also show that different nonlocal quantum correlations have been characterized by the physical property, i.e, complete local description of one of the subsystems, which is quantified by the local uncertainty relation conditioned on the outcomes of subsystems. The violation of local uncertainty relation confirms the nonlocal correlation between subsystems. When increasing the uncertainty of the condition by restricting the communication between two parties, local uncertainty relation detects stronger form of nonlocal quantum correlation. Therefore, uncertainty of local description of one of the subsystems can be interpreted as nonlocal correlation between subsystems. As an example, in experiment, we have tested the uncertainty of local descriptions and the quantum correlation of subsystems prepared in the bipartite Werner states. The framework presented in this work may open new possibilities for interpretation of quantum correlation with respect to other fundamental properties of the multipartite systems.

## Supplementary Information

Supplementary Information.

## Data Availability

The main data supporting the finding of this study are available within the article. Additional data can be provided upon request.
