# Supplementary material for: Physical interpretation of nonlocal quantum correlation through local description of subsystems
Source: Sci Rep. 2022 Sep 30;12:16400. doi: 10.1038/s41598-022-17540-1 (PMC9525634; doi:10.1038/s41598-022-17540-1)
Supplement: Supplementary file 1 — Supplementary Information. [file 41598_2022_17540_MOESM1_ESM.pdf]

Physical interpretation of nonlocal quantum correlation through local description of subsystems

Tanumoy Pramanik,[1,2,*] Xiaojiong Chen,[1] Yu Xiang,[1] Xudong Li,[1] Jun Mao,[1] Jueming Bao,[1] Yaohao Deng,[1] Tianxiang Dai,[1] Bo Tang,[3] Yan Yang,[3] Zhihua Li,[3] Qihuang Gong,[1,2,4,5,6] Qiongyi He,[1,2,4,5,6] and Jianwei Wang[1,2,4,5,6]

[1] *State Key Laboratory for Mesoscopic Physics, School of Physics, Peking University, Beijing, 100871, China*
[2] *Beijing Academy of Quantum Information Sciences, Beijing 100193, China*
[3] *Institute of Microelectronics, Chinese Academy of Sciences, Beijing 100029, China*
[4] *Frontiers Science Center for Nano-optoelectronics & Collaborative Innovation Center of Quantum Matter, Peking University, Beijing, 100871, China*
[5] *Collaborative Innovation Center of Extreme Optics, Shanxi University, Taiyuan 030006, Shanxi, China*
[6] *Peking University Yangtze Delta Institute of Optoelectronics, Nantong 226010, Jiangsu, China*
(Dated: November 27, 2021)

* Corresponding author

I. COMPLETE LOCAL DESCRIPTION OF QUANTUM SYSTEM

In the seminal work [1], Einstein et al. discussed about incompleteness of quantum theory in the presence of entanglement. J. Bell extended this work by considering quantum theory supplemented by the hidden variable, say, λ distributed according to $\{P(\lambda)\}$ where the restriction on λ are $P(\lambda) \geq 0$ and $\sum_\lambda P(\lambda) = 1$ [2]. They showed that in the presence of entanglement between the system A and B,

$$|\psi\rangle_{AB} = \frac{|01\rangle - |10\rangle}{\sqrt{2}}, \tag{1}$$

quantum theory can be incomplete [1, 2]. The incompleteness occurring from the violation of Bell inequality by the correlation $\mathcal{P}$ of Eq. (3) is known as Bell nonlocality, and the corresponding correlation is known as Bell nonlocal correlation. There are other well-known nonlocal quantum correlations, e.g., entanglement and steering. These nonlocal correlations have been explained with respect to trusting-untrusting scenarios [3, 4].

In this work, we have revisited the idea introduced by Einstein [1] et al. and Bell [2] considering the following question : when the systems A and B are quantumly correlated, is there any *complete local description* of the subsystems? Interestingly, if two systems A and B are in the separable state of

$$\rho_{AB}^{\text{LHS}} = \sum_i p_i \rho_i^A \otimes \rho_i^B, \tag{2}$$

where ρ_i^A (ρ_i^B) is Alice's (Bob's) local state, $p_i \geq 0$, and $\sum_i p_i = 1$, individual system A (B) has *complete local description*, $\{p_i,\, \rho_i^B\}$ ($\{p_i,\, \rho_i^A\}$), i.e., systems A and B do not share quantum correlation. For the verification of the nonlocal correlation of the bipartite state ρ_{AB}, let us consider the following game. In this game, Alice prepares two quantum systems A and B in the an entangled state ρ_{AB}, and sends the system B to Bob. Bob thinks that Alice may cheat him by preparing the system A and B in the separable state of the form of Eq. (2). For the verification of nonlocal correlation of the shared state ρ_{AB}, Bob asks Alice to reduce his uncertainty about the state of the system B for the measurement of observables chosen randmonly from the set of non-commuting observables $\{\mathcal{B}_j\}$. Therefore, communication of k-cbit (classical-bit) is required from Bob to Alice. According to Bob's information, Alice measures $\mathcal{A}_i$ on her system and communicates the measurement outcome a and the choice of observables $\mathcal{A}_i$. From the information $\{a,\, \mathcal{A}_i\}$, Bob constructs the joint probability distribution

$$\mathcal{P} = \left\{P(a_{\mathcal{A}_i}, b_{\mathcal{B}_j}; \rho_{AB}) = Tr[\left(\Pi_a^{\mathcal{A}_i} \otimes \Pi_b^{\mathcal{B}_j}\right).\rho_{AB}]\right\} \tag{3}$$

and checks the uncertainty of the *condition* characterized by $V_k(a, b|i, j)$. $V_k(a, b|i, j)$ corresponds to the desired correlation between outcomes a and b for the measurement of observables $\mathcal{A}_i$ and $\mathcal{B}_j$, and $k \in 1,\, 2,\, 3$ corresponds to three different conditions. The *local uncertainty* relation associated with the condition $V_k(a, b|i, j)$ becomes

$$\mathcal{F}_k^n = \sum_{i,j=0}^{n-1} \sum_{a,b=0}^{1} V_k(a, b|i, j) P(a_{\mathcal{A}_i, b_{\mathcal{B}_j}}|\rho_{AB}) \leq \mathcal{C}_k^n, \tag{4}$$

where n is the number of observables chosen by Alice and Bob, $k \in \{1,\, 2,\, 3\}$ corresponds three different condition for discrimination of three different nonlocal correlations (i.e., entanglement, steering and Bell nonlocal correlation). The upper bound $\mathcal{C}_k^n$ is obtained by maximizing $\mathcal{F}_k^n$ over the shared state ρ_{AB}^{LHS} and Alice's all possible strategies. The violation of the inequality (4) implies that the shared state ρ_{AB} can not be written in the form of ρ_{AB}^{LHS} of Eq. (2) and Bob system does not have *complete local description* under considered $V_k(a, b|i, j)$. As a result, Bob validates the nonlocal correlation of the shared state ρ_{AB}.

II. INCOMPLETE LOCAL-DESCRIPTION OF THE SYSTEM B DUE TO THE PRESENCE OF ENTANGLEMENT

LHS_1^n : Fig. (1) describes the schematic diagram to certify the entanglement of the shared state ρ_{AB}. For the verification of entanglement of the shared state ρ_{AB}, Bob checks the uncertainty of the condition

$$V_1(a,\, b|i,\, j) = (-1)^{a+b} \delta_{\mathcal{A}_i,\, \mathcal{B}_j}\, \delta_{a\, \bar{b}}, \tag{5}$$

where $\delta_{\mathcal{A}_i,\, \mathcal{B}_j} = 1$ and $\delta_{a\, \bar{b}} = 1$ only for $\mathcal{A}_i = \mathcal{B}_j$ and $a = \bar{b} = b \oplus 1$, respectively. In simple words, Bob checks the uncertainty of anti-correlation of their outcomes, $a \oplus b = 1$ when they measure same observable $\mathcal{A}_i = \mathcal{B}_j$ on their respective systems. As a result, Bob's needs to send the information of $\{b,\, \mathcal{B}_j\}$ to Alice, and it requires 2-cbit ($\log_2^{k=4}$,

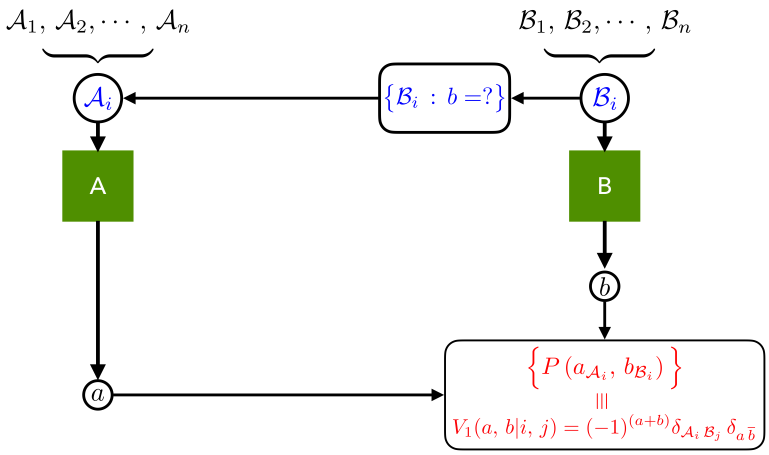

FIG. 1. Bob's strategy to verify whether the experimentally observed correlation $\{P(a_{\mathcal{A}_i}, b_{\mathcal{B}_i})\}$ infers the complete local description of the system B. Entanglement is confirmed when $\{P(a_{\mathcal{A}_i}, b_{\mathcal{B}_i})\}$ cannot be explained by the condition $V_1(a, b|i, j) = (-1)^{a+b}\delta_{\mathcal{A}_i, \mathcal{B}_j}\,\delta_{a\,\bar{b}}$.

corresponding to the four different combinations of two different outcomes and two measurement settings) or 2.58-cbit ($\log_2^{k=6}$, corresponding to the six different combinations of two different outcomes and three measurement settings) for the choice of two measurement settings or three measurement seetings, respectively. In the case of verification condition, $V_1(a, b|i, j)$ of Eq. (5), the inequality (4) becomes

$$\mathcal{F}_1^n = \sum_{i=0}^{n-1} P(0_{\mathcal{A}_i}, 1_{\mathcal{B}_i}) + P(1_{\mathcal{A}_i}, 0_{\mathcal{B}_i}) \leq \max_{\rho_{Ab}^{\rm LHS}} [\mathcal{F}_1^n] = \mathcal{C}_1^n \tag{6}$$

where maximization is taken over all possible choices of $\rho_{AB}^{\rm LHS}$. For the choice of two measurement settings, say, $\mathcal{A}_1 = \mathcal{B}_1 = \sigma_x$ and $\mathcal{A}_2 = \mathcal{B}_2 = \sigma_x$, the upper bound of the inequality (6) becomes $\mathcal{C}_1^{n=2} = 1$. In the case of three measurement settings, $\mathcal{A}_1 = \mathcal{B}_1 = \sigma_x$, $\mathcal{A}_2 = \mathcal{B}_2 = \sigma_y$, $\mathcal{A}_3 = \mathcal{B}_3 = \sigma_z$, $\mathcal{C}_1^{n=3} = 2$. In this scenario, the optimal cheating strategy for Alice corresponds to $\mathcal{F}_1^n = \mathcal{C}_1^n$ and the system B has complete local description $\in \{|0\rangle, |1\rangle, \sqrt{\alpha}|0\rangle + \sqrt{1-\alpha}|1\rangle\}$. Note here that the above complete local description is not unique. The inequality (6) is the *local uncertainty relation*, where '*local*' signifies that uncertainty relation is satisfied by the quantum systems having *complete local description* as shown in the Eq. (2). From the violation of the uncertainty relation (6), Bob validates the nonlocal correlation between system B and A and corresponding nonlocal correlation called entanglement [5–7]. The violation of the inequality (6) is the necessary criterion for verification of entanglement. Alice's knowledge about Bob's strategy makes the criterion (6) weakest. As a result, entanglement is the weakest nonlocal correlation.

III. INCOMPLETE LOCAL DESCRIPTION OF THE SYSTEM B DUE TO THE PRESENCE OF STEERABILITY

LHS_2^n : Fig. (2) describes the scenario to verify the steerability of the shared state ρ_{AB}. To verify steerability of the shared state ρ_{AB}, Bob checks the uncertainty of the condition

$$V_2(a, b|i, j) = (-1)^{a+b}\delta_{i,j}, \tag{7}$$

which corresponds to Bob's residual uncertainty associated with the measurement of non-commuting observables $\{B_i\}$ from the knowledge of $\{a, \mathcal{A}_i\}$. For consideration of $V_2(a, b|i, j)$ of Eq. (7), the uncertainty relation (4) becomes

$$\mathcal{F}_2^n = \sum_{i=0}^{n-1} |\langle \mathcal{A}_i\, \mathcal{B}_i \rangle| \leq C_2^n = \max_{\{\mathcal{A}_i\}, \rho_{AB}^{\rm LHS}} [\mathcal{F}_2^n], \tag{8}$$

where maximization is taken over all possible choices of $\{\mathcal{A}_0, \mathcal{A}_1, \cdots, \mathcal{A}_{n-1}\}$ and $\rho_{AB}^{\rm LHS}$. When Bob randomly choose observable from the set of two (three) non-commuting observables, say, $\{\sigma_x, \sigma_z\}$ ($\{\sigma_x, \sigma_y, \sigma_z\}$) the upper bound $C_2 = \sqrt{2}$ ($C_3 = \sqrt{3}$) occurs when Bob's system B has a complete local description by the eigenstates of observables $\frac{\sigma_x \pm \sigma_z}{\sqrt{2}}$ ($\frac{\sigma_x \pm \sigma_y \pm \sigma_z}{\sqrt{3}}$). The violation of the inequality (8) implies nonlocal correlation between Bob's system and Alice's

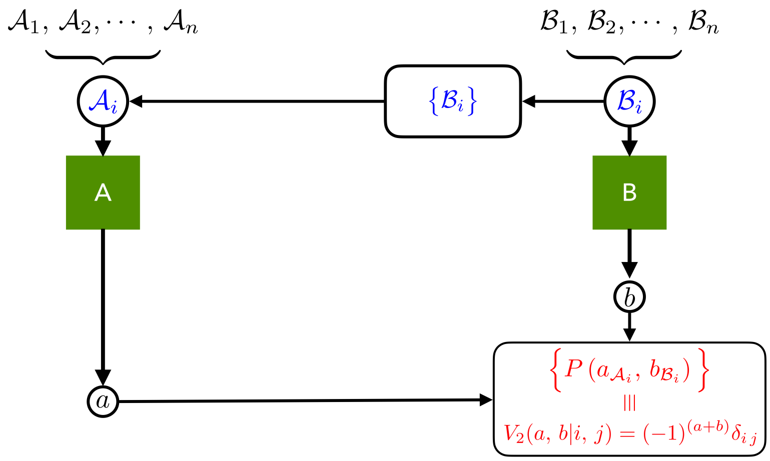

FIG. 2. Bob's strategy to verify whether the experimentally observed correlation $\{P(a_{\mathcal{A}_i}, b_{\mathcal{B}_i})\}$ corresponds to the complete local description of the system B. Steerability is certified when $\{P(a_{\mathcal{A}_i}, b_{\mathcal{B}_i})\}$ cannot be explained by the condition $V_2(a, b|i, j) = (-1)^{a+b}\delta_{i,j}$.

system, and it is known as steering [8–10]. Note here that the uncertainty relation (6) is weaker form of the uncerainty relation (8) as inequality (6) deals with the uncertainty of $a \oplus b = 1$ while inequality (8) corresponds to the uncertainty of both the condition $a \oplus b = 1$ and $a \oplus b = 0$. Therefore, the violation of uncertainty relation (8) indicates a stronger nonlocal correlation than the nonlocal correlation characterized by the uncertainty relation (6). Using $\langle \mathcal{A}_i \mathcal{B}_i \rangle = Ps\,(\mathcal{A}_i, \mathcal{B}_i) - Pd\,(\mathcal{A}_i, \mathcal{B}_i)$ (where $Ps\,(\mathcal{A}_i, \mathcal{B}_i) = P(0_{\mathcal{A}_i}, 0_{\mathcal{B}_i}) + P(1_{\mathcal{A}_i}, 1_{\mathcal{B}_i})$) and $Pd\,(\mathcal{A}_i, \mathcal{B}_i) + Ps\,(\mathcal{A}_i, \mathcal{B}_i) = 1$, inequality 8 becomes

$$|2\sum_{i=1}^{n} Pd\,(\mathcal{A}_i, \mathcal{B}_i) - n| \leq C_n. \tag{9}$$

Violation of the above inequality indicate the violation of the inequality (6), but the reverse is not true. Therefore, all steerable states are entangled and steerblity is stronger form of nonlocal correlation than entanglement.

Note that the violation of inequality (8) is the necessary criterion for steerability, and it becomes more efficient to capture steerability of the given state with increment of the number of measurement settings, n [8, 9]. But if inequality (8) is satisfied, unsteerability can not be concluded. The unsteerability of the given state can be verified from the sufficient criteria [11], but it does not tell about steerability.

IV. INCOMPLETE LOCAL-DESCRIPTION OF THE SYSTEM B DUE TO THE PRESENCE OF BELL NONLOCLALITY

LHS_3^n : Fig. (3) describes the scenario to validate the Bell nonlocal correlation of the shared state ρ_{AB}. In the case of Bell nonlocality, Bob keeps secret the information of the observable $\mathcal{B}_i$ going to be measured on the system B. In the case of two measurement settings, he checks the uncertainty of the condition

$$V_3(a, b|i, j) = (-1)^{(a+b+ij)}, \tag{10}$$

which corresponds the winning condition of CHSH game [2, 12, 13]. The uncertainty relation (4) for the choice of $V_2(a, b|i, j)$ of Eq. (10) becomes

$$\mathcal{F}_3^2 \leq \max_{\{\mathcal{A}_i\},\, \rho_{AB}^{\text{LHS}}} \left[\mathcal{F}_3^2\right] = 2, \tag{11}$$

where $\mathcal{F}_3^2 = |\langle \mathcal{A}_0\,(\mathcal{B}_0 + \mathcal{B}_1)\rangle + \langle \mathcal{A}_1\,(\mathcal{B}_0 - \mathcal{B}_1)\rangle|$ corresponds to Bob's residual uncertainty of the set of non-commuting observables $\{\mathcal{B}_0, \mathcal{B}_1\}$ from the knowledge of individual $\{a, \mathcal{A}_i\}$ and $i \in 0, 1$. The above inequality is the necessary and sufficient criterion for Bell nonlocality for 2-measurement-setting and 2-outcome scenario [14]. The equality $BI_2 = 2$ occurs when Bob's system has a complete local description by $\{|0\rangle, |1\rangle, \sqrt{\alpha}|0\rangle + \sqrt{1-\alpha}|1\rangle\}$. Note that the above local description is also not unique.

Recent developments improve Bell inequalities with n number of measurement settings per side [15–18]. Among them Bell inequality given in the Ref. [15] is the inequivalent class of Bell-CHSH inequality and it can detect those

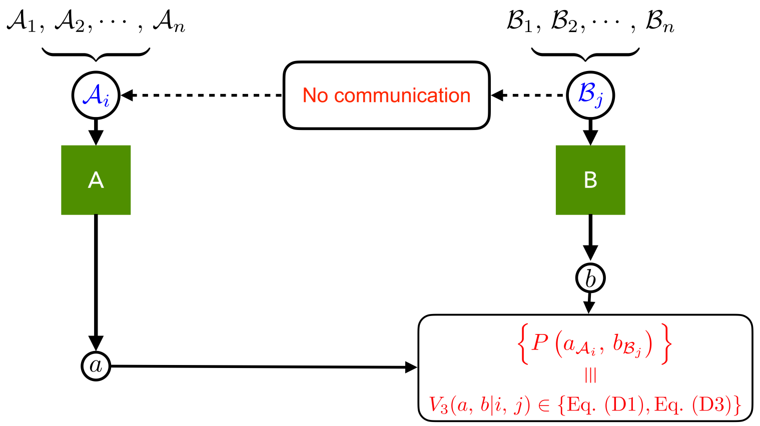

FIG. 3. Bob's strategy to verify whether the experimentally observed correlation $\{P(a_{\mathcal{A}_i},\, b_{\mathcal{B}_i})\}$ corresponds to the complete local description of the system B. Bell nonlocality is verified when $\{P(a_{\mathcal{A}_i},\, b_{\mathcal{B}_i})\}$ cannot be explained by the condition $V_3(a,\, b|i,\, j) \in \{\text{Eq.(10), Eq.(12)}\}$.

entangled state which is Bell-CHSH local. Particularly, for the Werner state, Bell inequality with 465 measurement settings per side has been developed [16] which can show Bell nolocality of Werner state for $0.7056 < p \leq 1$. This inequality [16] gives the tightest Bell violation for Werner state after Bell-CHSH inequality. Therefore, Bell-CHSH inequality (11) works better than the existing 3-setting Bell inequality. Here, for three measurement settings, we consider Bell inequality given in the Ref. [15] as an example. Bob checks the following condition

$$V_2(a,\, b|i,\, j) = (-1)^{a\oplus b=\max[0,i.j-1]}(1-\delta_{i,3}\delta_{j,3}) + (-1)^a\,(1-\delta_{i,3}) - (-1)^b(1-\delta_{j,3}). \tag{12}$$

Under this condition, the inequality (4) becomes

$$\mathcal{F}_3^3 \leq \max_{\{\mathcal{A}_i\},\,\rho_{AB}^{\text{LHS}}} \left[\mathcal{F}_3^2\right] = 4, \tag{13}$$

where $\mathcal{F}_3^3 = |\langle \mathcal{A}_1\,(\mathrm{I}+\mathcal{B}_1\rangle + \mathcal{B}_2 + \mathcal{B}_3\rangle) + \langle \mathcal{A}_2\,(\mathrm{I}+\mathcal{B}_1+\mathcal{B}_2-\mathcal{B}_3)\rangle + \langle \mathcal{A}_3\,(\mathcal{B}_1-\mathcal{B}_2)\rangle - \langle \mathcal{B}_1\rangle - \langle \mathcal{B}_2\rangle|$, and Bob chooses observables randomly from the set $\{\mathcal{B}_1 = \sigma_z,\, \mathcal{B}_2 = \sin(\frac{\pi}{3})\sigma_x + \cos(\frac{\pi}{3})\sigma_z,\, \mathcal{B}_3 = \sin(\frac{2\pi}{3})\sigma_x + \cos(\frac{2\pi}{3})\sigma_z\}$. In this scenario, $|0\rangle$ becomes one of the complete local description of the system B corresponding to $\mathcal{F}_3^3 = 4$. The inequality (13) detects Bell nonlocality of the Werner state (17) for $p > 0.8$, whereas the inequlality (11) confirms Bell nonlocality for $p > 1/\sqrt{2}$.

Bell-CHSH inequality (11) works better for the two-qubit Werner state than existing Bell inequalities with three measurement settings and two outcomes scenario [15–18]. Note that, mathematically, inequality (11) can be written

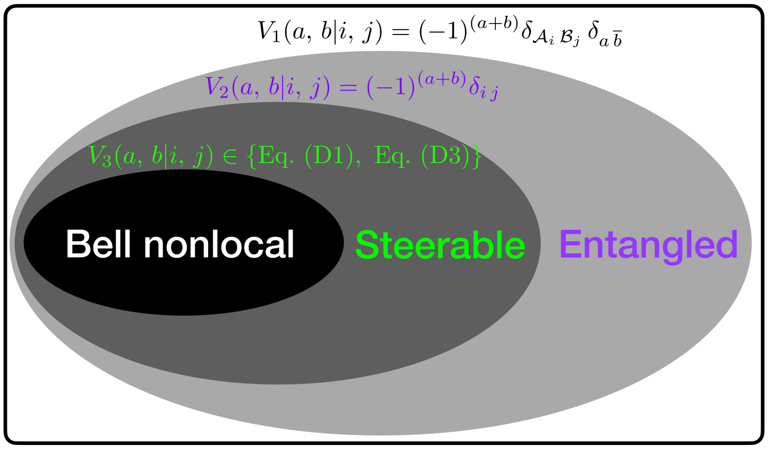

FIG. 4. Different nonlocal quantum correlations. Bell nonlocal correlation is the strongest form of all nonlocal correlations, and entanglement is the weakest while steering lies between Bell nonlocal correlation and entanglement.

as

$$BI_2 = |\langle \mathcal{A}'_1 \mathcal{B}_1 \rangle + \langle \mathcal{A}'_2 \mathcal{B}_2 \rangle| \leq \sqrt{2}, \tag{14}$$

where $\mathcal{A}_1 = \frac{\mathcal{A}_1 + \mathcal{A}_2}{\sqrt{2}}$ and $\mathcal{A}_2 = \frac{\mathcal{A}_1 - \mathcal{A}_2}{\sqrt{2}}$. Therefore, violation of inequality (11) implies the violation of inequality (8) while the reverse is not true. Therefore, all Bell nonlocal states are steerable. Unlike steerability, Bell nonlocality includes the uncertainty of choice of Bob's observables from the set $\{\mathcal{B}_1, \mathcal{B}_2, \cdots, \mathcal{B}_n\}$ which makes Bell nonlocal correlation as a strongest form nonlocal correlation than steerability and entanglement. A complete picture of all the correlations can be viewed as in Fig. (4), which represents that Bell nonlocal correlation forms a subset of both steering and entanglement, while steering is itself a subset of entanglement.

V. EXPERIMENTAL SETUP AND DEMONSTRATION

Fig. (3) (in the main text) represents the experimental setup designed on a silicon photonic chip which is used to demonstrate different quantum correlations from the violation of different forms of uncertainty relations (6, 8, 11). Here, a pair of path-encoded entangled photons in the state $|\psi^+\rangle$ $(= (|00\rangle + |11\rangle)/\sqrt{2})$ are generated via spontaneous four-wave mixing (SFWM) process, by pumping a continuous wavelength laser at 1550.12nm on two spiral waveguides (single photon-pair sources). The signal photon (1545.31nm, shown in red) is assumed to be system A (belonging to Alice) while the idler photon (1554.91nm, shown in green) is assumed to be system B (belonging to Bob). The expectation value $\langle \mathcal{A}\mathcal{B} \rangle_{|\psi^+\rangle}$,

$$\langle \mathcal{A}\mathcal{B} \rangle_{|\psi^+\rangle} = \langle \psi^+ | \mathcal{A} \otimes \mathcal{B} | \psi^+ \rangle \tag{15}$$

can be experimentally observed from coincidence detection

$$P(a_{\mathcal{A}}, b_{\mathcal{B}}) = \frac{C(a_{\mathcal{A}}, b_{\mathcal{B}})}{\sum_{\{a,b\}=0}^{1} C(a_{\mathcal{A}}, b_{\mathcal{B}})}, \tag{16}$$

for the measurement of $\mathcal{A}$ and $\mathcal{B}$ on system A and B prepared in the state $|\psi^+\rangle$, respectively. Here, $C(a_{\mathcal{A}}, b_{\mathcal{B}})$ represents coincident counts for the outcome a and b .

In experiment, direct generation of two-qubit Werner state ρ_W is still challenging as it requires the mixture of Bell

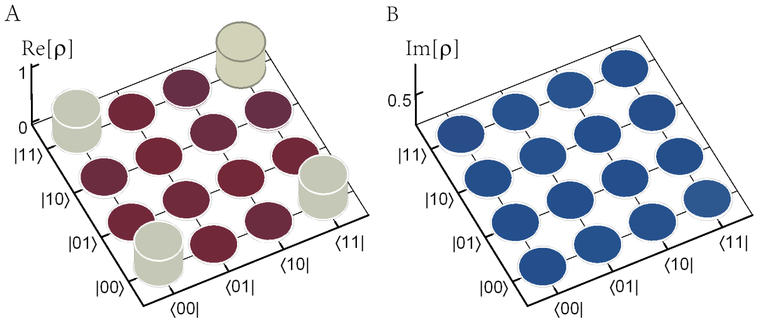

FIG. 5. Quantum state tomography. The real(A) and imaginary(B) part of density matrix, for the target state $|\psi^+\rangle = \frac{1}{\sqrt{2}}(|00\rangle + |11\rangle)$, is plotted with a fidelity of 0.951 ± 0.096. Over-complete quantum state tomography is used to reconstruct the experimental state, which requires 9 different measurement settings and a total of 36 local projection measurement basis combinations.

state and maximal incoherent state, which can be described by

$$\rho_W = p\rho_{|\phi^-\rangle} + (1-p)\,\frac{\mathrm{I}\otimes\mathrm{I}}{4}$$
$$= \frac{\alpha\,(\mathrm{I}\otimes\sigma_{\mathrm{x}})\,\rho_{|\psi^+\rangle}\,(\mathrm{I}\otimes\sigma_{\mathrm{x}}) + \beta\,(\mathrm{I}\otimes\,\sigma_{\mathrm{y}})\,\rho_{|\psi^+\rangle}\,(\mathrm{I}\otimes\,\sigma_{\mathrm{y}}) + \gamma\,\rho_{|\psi^+\rangle} + \delta\,(\mathrm{I}\otimes\sigma_{\mathrm{z}})\,\rho_{|\psi^+\rangle}\,(\mathrm{I}\otimes\sigma_{\mathrm{z}})}{\alpha+\beta+\gamma+\delta}, \tag{17}$$

where $\frac{\alpha}{\alpha+\beta+\gamma+\delta} = \frac{1+3p}{4}, \beta = \delta = \gamma = \frac{1-p}{4}(\alpha+\beta+\gamma+\delta)$, $\rho_{|\phi^-\rangle}$ and $\rho_{|\psi^+\rangle}$ are the density matrix of the states $|\phi^-\rangle$ $(= (|01\rangle - |10\rangle)/\sqrt{2})$ and $|\psi^+\rangle$, respectively. The expectation value $\langle\mathcal{A}\mathcal{B}\rangle_{\rho_W}$ for the Werner state ρ_W has been experimentally realized from the statistical mixing of identity (I) and Pauli rotations $(\sigma_x, \sigma_y,\ \sigma_z)$ on observable $\mathcal{B}$ as given by

$$\langle\mathcal{A}\mathcal{B}\rangle_{\rho_w} = \frac{\alpha\langle\mathcal{A}\mathcal{B}_x\rangle_{|\psi^+\rangle} + \beta\langle\mathcal{A}\mathcal{B}_y\rangle_{|\psi^+\rangle} + \gamma\langle\mathcal{A}\mathcal{B}\rangle_{|\psi^+\rangle} + \delta\langle\mathcal{A}\mathcal{B}_z\rangle_{|\psi^+\rangle}}{\alpha+\beta+\gamma+\delta}, \tag{18}$$

where $\mathcal{B}_i = \sigma_i\,\mathcal{B}\,\sigma_i,\ i \in \{x,\,y,\,z\}$. The $\langle\mathcal{A}\mathcal{B}\rangle_{\rho_W}$ is calculated from the experimentally observed data for $p = 0.0, 0.2, 0.4, 0.6, 0.8, 1.0$, and the corresponding mixing weights $\{\alpha,\ \beta\}$ are

$$\begin{aligned} &p{=}\ 0.0 \to \{\alpha=5, \beta=5\}, && p=0.6 \to \{\alpha=14, \beta=2\},\\ &p{=}\ 0.2 \to \{\alpha=8, \beta=4\}, && p=0.8 \to \{\alpha=17, \beta=1\},\\ &p{=}\ 0.4 \to \{\alpha=11, \beta=3\}, && p=1.0 \to \{\alpha=20, \beta=0\}. \end{aligned} \tag{19}$$

For example, $p = 0.0$ is calculated from the 5 sets of data of each $\langle A\mathcal{B}_i\rangle_{|\psi^+\rangle}$. In the experiment, the Bell state $|\psi^+\rangle$ has been generated with fidelity 0.951 ± 0.096, and the details of quantum state tomography (QST) is shown in the Fig.5. The error bars in terms of standard deviation have been calculated from the 20 sets of data, which result in the order of 10^{-2} for entanglement, steering and Bell nonlocality.

[1] A. Einstein, D. Podolsky, and N. Rosen, *Can Quantum-Mechanical Description of Physical Reality Be Considered Complete?*, Phys. Rev. **47**, 777 (1935).

[2] J. S. Bell, *On the Einstein Podolsky Rosen paradox*, Physics **1**, 195 (1964).

[3] H. M. Wiseman, S. J. Jones, and A. C. Doherty, *Steering, Entanglement, Nonlocality, and the Einstein-Podolsky-Rosen Paradox*, Phys. Rev. Lett. **98**, 140402 (2007).

[4] S. J. Jones, H. M. Wiseman, and A. C. Doherty, *Entanglement, Einstein-Podolsky-Rosen correlation, Bell nonlocality, and steering*, Phys. Rev. A **76**, 052116 (2007).

[5] M. Berta, M. Christandl, R. Colbeck, J. M. Renes, and R. Renner, *The uncertainty principle in the presence of quantum memory*, Nature Phys. **6**, 659 (2010).

[6] C. Li, J. Xu, X. Xu, K. Li, and G. -C. Guo, *Experimental investigation of the entanglement-assisted entropic uncertainty principle*, Nature Phys. **7**, 752 (2011).

[7] T. Pramanik, P. Chowdhury, and A. S. Majumdar, *Fine-Grained Lower Limit of Entropic Uncertainty in the Presence of Quantum Memory*, Phys. Rev. Lett. **110**, 020402 (2013).

[8] D. J. Saunders, S. J. Jones, H. M. Wiseman, and G. J. Pryde, *Experimental EPR-steering using Bell-local states*, Nature Phys. **6**, 845 (2010).

[9] A. J. Bennet, D. A. Evans, D. J. Saunders, C. Branciard, E. G. Cavalcanti, H. M. Wiseman, and G. J. Pryde, *Arbitrarily Loss-Tolerant Einstein-Podolsky-Rosen Steering Allowing a Demonstration over 1 km of Optical Fiber with No Detection Loophole*, Phy. Rev. X **2**, 031003 (2012).

[10] T. Pramanik, M. Kaplan, and A. S. Majumdar, *Fine-grained Einstein-Podolsky-Rosen-steering inequalities*, Phys. Rev. A **90**, 050305(R) (2014).

[11] J. Bowles, F. Hirsch, M. T. Quintino, and N. Brunner, *Sufficient criterion for guaranteeing that a two-qubit state is unsteerable*, Phys. Rev. A **93**, 022121 (2016).

[12] J. F. Clauser, M. A. Horne, A. Shimony and R. A. Holt, *Proposed Experiment to Test Local Hidden-Variable Theories*, Phys. Rev. Lett. **23**, 880 (1969).

[13] J. Oppenheim, and S. Wehner, *The Uncertainty Principle Determines the Nonlocality of Quantum Mechanics*, Science **330**, 1072 (2010).

[14] R. Horodecki, P. Horodecki and M. Horodecki, *Violating Bell inequality by mixed states: necessary and sufficient condition*, Phys. Lett. A 200, **340** (1995).

[15] D. Collins, and N. Gisin, *A relevant two qubit Bell inequality inequivalent to the CHSH inequality*, J. Phys. A: Math. Gen. **37** 1775 (2004).

[16] T. Vertesi, *More efficient Bell inequalities for Werner states*, Phys. Rev. A **78**, 032112 (2008).

[17] A. Salavrakos, R. Augusiak, J. Tura, P. Wittek, A. Acin, and S. Pironio, *Bell Inequalities Tailored to Maximally Entangled States*, Phys. Rev. Lett. **119**, 040402 (2017).

[18] K. F. Pál and T. Vértesi, *Maximal violation of a bipartite three-setting, two-outcome Bell inequality usinginfinite-dimensional quantum systems*, Phys. Rev. A 82, 022116 (2010).
